# Management of Verticillium Wilt of Avocado Using Tolerant Rootstocks

**DOI:** 10.3390/plants9040531

**Published:** 2020-04-20

**Authors:** Amnon Haberman, Leah Tsror (Lahkim), Silit Lazare, Marina Hazanovsky, Sara Lebiush, Isaac Zipori, Amnon Busatn, Eli Simenski, Arnon Dag

**Affiliations:** 1 Gilat Research Center, Agricultural Research Organization, M.P. Negev 8528000, Gilat, Israelmatabsor@volcani.agri.gov.il (I.Z.); 2 CSIRO Agriculture and Food, Locked Bag 2, Glen Osmond, South Australia 5065, Australia; 3 Desert Agro-Research Center, Ramat Negev Works Ltd., M.P. Halutza 8551500, Israel; 4 Neva-Team Agricultural Applications Ltd., Moshav Carmel, M.P. Har Hebron 9040400, Israel

**Keywords:** *Verticillium dahlia*, *Persea Americana*, tolerance, resistant, disease

## Abstract

The global avocado industry is growing, and farmers are seeking to expand their plantations. However, many lands suitable for avocado planting were previously cultivated with hosts of the soil-borne fungal pathogen *Verticillium dahliae*, which is the causal agent of Verticillium wilt (VW). VW can seriously impair avocado orchards, and therefore, planting on infested soil is not recommended. The use of different rootstock types allows avocado cultivation in various regions with diverse biotic and abiotic constraints. Hence, we tested whether genetic variance among rootstocks may also be used to manage avocado VW. Six hundred trees, mostly Hass and some Ettinger, grafted on 23 selected rootstocks were evaluated for five years in a highly *V. dahliae*-inoculated plot for VW symptoms, fungal infection, and productivity. The selected rootstocks displayed a significant variation related to VW tolerance, and productive avocado rootstocks with potential VW tolerance were identified. Moreover, the rootstock productivity appears to correlate negatively to the susceptibility level. In conclusion, planting susceptible rootstocks (e.g., VC66, VC152, and VC26) in infested soil increases the likelihood of massive tree loss and low productivity. Whereas, tolerant rootstocks (e.g., VC804 and Dusa) may restrict VW and enable avocado cultivation on infested soils.

## 1. Introduction

The global avocado (*Persea americana* Mill.) market is constantly growing, with the harvested area tripled in the last two decades (FAOSTAT data). In accordance, avocado growers are constantly seeking to expand their orchards. However, many agricultural lands suitable for avocado were previously used for the cultivation of crops that are hosts of the fungal soil-borne pathogen *Verticillium dahliae*. The wide list of hosts include over 200 plant species [1] with crops such as potato (*Solanum tuberosum* L.) [2], tobacco (*Nicotiana tabacum* L.) [3], cotton (*Gossypium hirsutum* L.; *Gossypium barbadense* L.) [4], mango (*Mangifera indica* L.) [5], and olive (*Olea europea* L.) [6]. As a result of crop rotation history, using susceptible hosts, many soils are suspicious for *V. dahliae* infestation [7].

The pathogen *V. dahliae* survives in the soil as microsclerotia for prolonged periods of over 20 years [8,9]. The microsclerotia germinate and invade the host roots [10]. Mycelium develops in the root cortex and enters the xylem vessels [11], where produced conidia spread up to the host canopy [10]. As a result, the plant vascular system may be clogged, which causes the disease Verticillium wilt (VW) [12,13]. Verticillium wilt is very difficult to control, due to a number of reasons: (1) the ability of the pathogen to produce and spread through microsclerotia, that survive in the soil for extended periods; (2) a vast spectrum of hosts, with varying symptoms; and (3) a lack of effective control measures [12,14].

**Figure 1:**
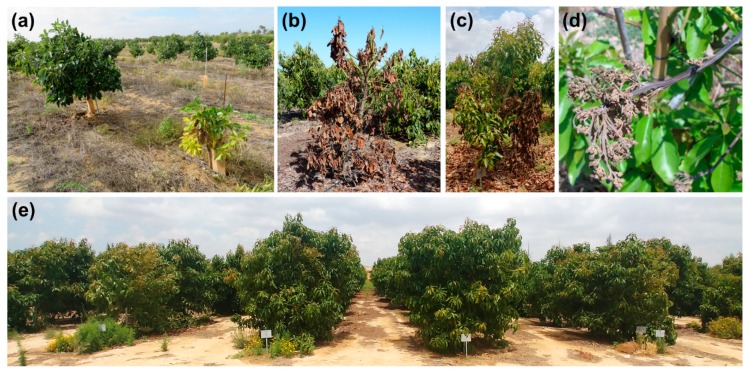
(**a**–**d**) Trees infected with *V. dahliae* showing Verticillium wilt (VW) symptoms. (**a**) Young trees, approximately a year and a half from planting. To the left, a healthy well-developed tree and to the right, a sick and degenerated tree. (**b**,**c**) Damage of VW, extensive branch dieback with drying leaves remaining attached to the branches. (**e**) Damage to an inflorescence, possibly due to VW. (**e**) Picture of the experimental plot.

VW disease symptoms on avocado include the sudden wilt and dieback of single or multiple branches, with the dead leaves typically remaining on the tree for several months (Figure 1a–d) [13,15]. Brown streaks can often be seen in the xylem of dying branches [15]. Young trees are more vulnerable and might collapse rapidly and die [16]. Scenarios of asymptomatic infection, in which mature trees might exhibit a latent impact of the disease, have been reported in olive and other host plants [17,18,19]. In these cases, the wilting of inflorescences and young vegetative growth may occur in the spring, followed by a later recovery during the summer, when temperatures increase above the optimum for the fungus [20]. As a result, many trees fail to fulfill their fruit production potential, and the orchard productivity is impaired without any definitive related symptoms.

Avocado trees are grown commercially in many regions worldwide, under different climates and soil properties [21]. This wide distribution is possible due to the availability of different rootstocks, and thus, most of the commercially grown avocado are grafted trees. The propagation of the rootstocks may be clonal or as seedlings [22]. The avocado variety ‘Hass’ is currently the most important scion variety [23], which accounted for 97% of the fresh avocado retail sales in the U.S. market in 2013–2017 [24]. However, due to the avocado synchronous dichogamy self-incompatibility flowering habit [25], many orchards will also include another scion variety to serve as a pollenizer.

Orchard profitability is highly dependent on choosing a suitable rootstock [22]. Rootstocks are used to address issues of soil salinity, acidity and aeration, tree size and vigor, and soil-borne pathogens, such as Phytophthora root rot (PRR) [22], which is caused by the oomycete pathogen *Phytophthora cinnamomi* [26]. PRR is the most economically significant disease of avocado in numerous regions, and host resistance is considered to be the best method to manage it [16]. Great efforts are invested in identifying PRR tolerance, and such rootstocks are available commercially [27,28,29,30].

The root system is suggested to play an important role in the tolerance mechanisms to VW [31]. In several plant species, grafting on resistant rootstocks is reported as a promising technique to manage VW [32,33,34]. For example, in olive cultivation, where VW is considered a major threat [6,35], grafting susceptible varieties onto resistant rootstock may provide effective management of the disease [36]. The avocado genetic pool available for rootstock selection is divided into three horticultural races, which are commonly referred to as Mexican, Guatemalan, and West Indian based on their presumed origin [37]. Some differences in the frequency of VW among avocado rootstocks were previously reported [38,39], suggesting that some genetic diversity for susceptibility to VW is available in avocado.

With no effective management measures to control VW, new avocado plantings on soils infested with *V. dahliae* are currently not recommended. However, due to the natural existing genetic variability in rootstocks, some level of tolerance or resistance is expected to be available, as obtained for PRR [27]. In the current study, we evaluated whether genetic variance may be used to overcome avocado VW. A total of 23 selected rootstocks (Table 1) were screened in a highly infested plot (Figure 1e) for VW tolerance and productivity. Identifying a VW-tolerant and highly productive rootstock will reduce the risk and may enable expanding avocado plantations into *V. dahliae*–infested areas.

## 2. Materials and Methods

### 2.1. Experimental Site

The experimental orchard was planted at Gilat Research Center (ARO), which is located in the northern Negev region of Israel. A region with a semi-arid climate characterized by a warm dry summer, cool winter, and average annual precipitation of 253 mm, normally falling between November and April. The orchard soil texture characterized as sandy loam, with a pH value of 8.2 and 11.5% calcium carbonate content. The soil composition is 50% sand, 35% silt, and 15% clay, with about 0.5% organic matter content. The orchard was irrigated and fertilized (fertigated) with a drip irrigation system twice a week, during the dry season (March to October) according to common commercial practices. The average annual irrigation dosage was 14,500 m^3^ ha^−1^, and the average fertilizer application levels were 206 kg N, 59 kg P_2_O_5_, 206 kg K_2_O, 0.9 kg Fe, 0.4 kg Mn, 0.2 kg Zn, 32 g Cu, and 24 g Mo per ha. Nutritional status was assessed annually by elements concentration in leaves [40].

### 2.2. Orchard Inoculation

For the inoculation with *V. dahliae*, potato dry stems infected with *V. dahliae* microsclerotia were collected from infested commercial plots and plowed into the soil in January 2011. Thereafter, to further increase the buildup of fungal inoculum in the soil, a relatively susceptible potato variety to VW (cv. Almera) was grown in the plot. At the end of the growing season, the dry potato stems had an 86% incidence of *V. dahliae* microsclerotia. Finally, before planting the trees, potato dry stems infected with *V. dahliae* microsclerotia were added to the planting pit.

### 2.3. Plant Material

The grafted trees were prepared during 2011 by Haskelberg Nurseries (Kfar Vitkin, Israel). The rootstocks selection for the screening trial was done mainly according to previous reports of some tolerance to PRR and the availability of propagation material. In June 2013, 600 avocado plants, 83% Hass and 17% Ettinger, grafted on 23 selected clonal or seedling rootstocks (Table 1) were planted in the inoculated plot. Planting spacings were four meters between rows and three meters within the row (4 m × 3 m; Figure 1e). The experiment was set up in five randomized blocks, with 20 plots in each block. The plots consisted of six adjacent trees of the same rootstock, five with a Hass scion and one with Ettinger that served as a pollenizer.

### 2.4. VW Symptoms Evaluation

VW symptoms were assessed by visual observation twice during September–November and twice during March–June when the fungus is most active under the growth conditions in the experimental orchard. Symptoms were defined as wilt and chlorosis, branch dieback with drying leaves remaining attached to the branches, and vascular discoloration (Figure 1a–d).

### 2.5. Verticillium Dahliae Isolation and Identification

The microbial examination of the symptomatic trees was conducted immediately after the assessment of the symptoms in the orchard. Samples of five diseased branches from each of the symptomatic trees were collected during the different seasons of the trial. Four segments, four cm in length, from each branch were surface sterilized with 0.3% HClO for seven min and rinsed with sterile water. Three pieces (2–5 mm long) from each segment were transferred to sorbose agar (SA) medium (0.2% *w/v* sorbose, 1.5% *w/v* agar, 100 ppm streptomycin), incubated at 25 °C in the dark, and examined after two weeks for the presence of *V. dahliae* by morphological characterization using a light microscope.

### 2.6. Productivity Assessment

Fruits were harvested and weighed separately per each tree during the four first seasons of fruit production (2015–2018). A sample of 10 fruits from each tree was weighed to calculate the fruit number per tree.

### 2.7. Data Analysis

**Figure 2:**
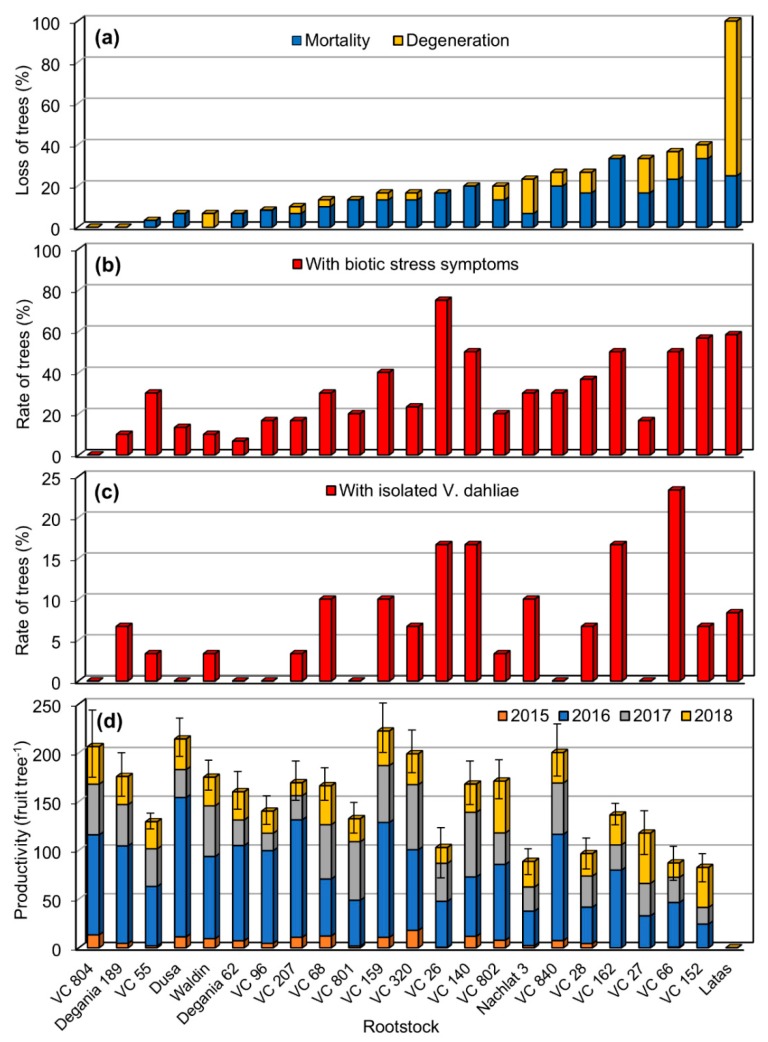
Trees were planted in 2013 and evaluated for five years. (**a**) Percentage of tree loss due to mortality and degeneration. (**b**) Percentage of trees with isolated *V. dahiae.* (**c**) Percentage of trees with VW symptoms (**d**) Trees productivity as the accumulative number of fruits harvested per tree in seasons 2015–2018. (**a**–**c**) Numbers are frequency in the population. (**d**) Numbers are mean values ± standard error of the mean (bars), statistical difference test results are presented in Table 2.

**Figure 3:**
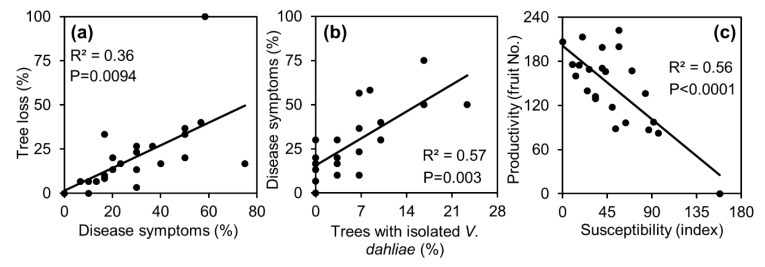
(**a**) Correlation between the rate of tree loss (Figure 2a) and the rate of disease symptoms (Figure 2b). (**b**) Correlation between the rate of disease symptoms (Figure 2b) and the rate of trees with isolated *V. dahliae* (Figure 2c). (**c**) Correlation between trees productivity (Figure 2d) and the susceptibility index (Table 2).

The susceptibility index (Table 2 and Figure 3b) was calculated by combining the values of the percentage of tree loss (Figure 2a) and the percentage of trees with biotic stress symptoms (Figure 2b; loss+symptoms). The rootstocks performance score value (in Table 2) was calculated by deducting the susceptibility index value from the productivity value (Table 2; productivity minus susceptibility). The productivity data presented are only of the Hass trees. Loss of trees, biotic stress symptoms, and *V. dahliae* isolation data are of the Hass and Ettinger trees combined. Statistical significance was analyzed by one-way analysis of variance (ANOVA), using JMP software (SAS Institute, Cary, NC, USA). Logit transformation [41] was applied to proportional data prior to ANOVA.

## 3. Results and Discussion

### 3.1. Loss of Trees

Loss of trees following planting is a major concern in the establishment of a new orchard. In such cases, the grower needs to replant the lost trees, resulting in additional expenses for the extra trees and delayed fruit production. In the current study, five years after planting (2018), the accumulated rate of mortality and degeneration of trees resulting in loss of trees was highly variable among the different rootstocks (Figure 2a). The highest rate of loss was observed in the Latas rootstock (100%). An additional six out of the 23 evaluated rootstocks, had substantial loss rates of more than 25% (VC152, VC66, VC27, VC162, VC28, and VC840). In contrast, in two rootstocks (VC804, Degania 189), none of the trees were lost and all developed and produced fruits.

The tree loss can be attributed to either the high infestation levels of *V. dahliae* in the orchard soil or the incompatibility of the rootstock to the orchard environmental conditions. The full loss (100%) of the Latas trees indicates that this rootstock is not suitable for the environmental conditions in the orchard, e.g., semi-arid climate or soil type (relatively high pH). Perhaps, the extreme impact on these trees is due to the combination of both the unsuitable conditions along with the high level of *V. dahliae* infestation. An interaction between *V. dahliae* and salinity was reported to cause enhanced VW symptoms and higher colonization levels in olive [42] and pistachio (*Pistacia vera* L.) [43]. Salinity is typical to soils in arid and semi-arid areas, such as the location of the trial plot [44].

### 3.2. Verticillium Wilt

Disease symptoms including typical wilt were observed on some of the trees (see Materials and Methods; Figure 1a–c). The rate of symptomatic trees was very diverse, ranging from 0% to 75% (Figure 2b). In six rootstocks (Latas, VC152, VC66, VC162, VC140, VC26), the incidence of disease symptoms was very high: 50%–80%. In contrast, none of the trees of the rootstock VC804 had any symptoms (Figure 2b). To assess whether the symptoms can be attributed to VW, symptomatic trees were tested for *V. dahliae* infection, and positive trees were detected in 16 out of the 23 rootstocks (Figure 2c). Overall, 44% of the tested trees for *V. dahliae* colonization were found positive.

The correlation between the rate of tree loss and the rate of disease symptoms (Figure 3a) shows that a considerable portion of the loss is associated with the symptoms, indicating that a significant portion of the tree loss may be due to the biotic stress. Nevertheless, some of the tree loss was probably due to other reasons that impact tree survival, mainly during the orchard establishment period. As a substantial proportion (44%) of the sampled symptomatic trees were indeed validated as *V. dahliae*-positive, it is likely that a significant portion of the tree loss was due to VW. This is also supported by the correlation between the rate of disease symptoms and positive isolation of *V. dahliae* (Figure 3b).

### 3.3. Productivity

Tree productivity was recorded as an accumulative number of fruits in four seasons (2015–2018). The productivity among the rootstocks was very different with the relatively higher productive rootstocks producing over 200 fruits tree^−1^ (e.g., VC159, Dusa, VC804, VC840) and lower productive rootstocks producing under 100 fruits tree^−1^ (e.g., VC152, VC66, VC28, Nachlat 3, VC26; Figure 2d). The fruit crop in the first production season (2015) was relatively very low; however, it demonstrated which rootstocks were earlier to start fruit production (e.g., VC320; Figure 2d). In the second production season (2016), yields increased significantly, followed by less productive seasons (2017–2018), which was probably due to the typical alternate bearing behavior of avocado [45].

The correlation between trees productivity and the susceptibility index (Figure 3c) suggests that in addition to the direct damage, VW also affects fruit production. In a mature orchard, where trees are less prone to collapse and symptoms may be less significant, a challenging scenario of ‘asymptomatic’ or ’latent’ VW may develop. This scenario is expressed in reduced fruit production without typical symptoms, indicating that the orchard is suffering from biotic stress. A possible explanation for this phenomenon may arise from the synchronization between avocado bloom and VW outbreak in the spring [19,20]. While avocado inflorescences are susceptible to high temperature and drought, water supply to flowers and fruitlets might be further challenged when infected by *V. dahliae*, which might significantly decrease the final number of fruits per tree. However, this assumption requires additional research.

In the distribution of trees for productivity and VW susceptibility, it is prominent that some rootstocks have both low susceptibility and high productivity. Deducing the susceptibility index value from the productivity (productivity minus susceptibility) provides a performance score that comprises the evaluation parameters (Table 2). Sorting the rootstocks by this score ranks them according to the overall performance. The variance in this score among the evaluated rootstocks is extremely high (Table 2), demonstrating the high genetic variability available and how it is translated to performance. In addition, this score facilitates the selection of the best performing rootstocks.

In general, due to the genetic uniformity, clonally propagated rootstocks are expected to be superior to seedling rootstocks [22]. Comparing the propagation method of the rootstocks (clonal or as seedlings), clonal rootstocks do not appear superior, as some of the seedling rootstocks scored very high in the relative performance (e.g., Degania 189; Table 2). The rootstocks performance under the high disease pressure in this study appear more quantitative rather than binary, supporting the notion that VW tolerance is a polygenic trait [46]. It was previously suggested that the avocado races differ in the susceptibility to VW and that Mexican rootstocks are less susceptible to VW than Guatemalan [39]; nevertheless, we could not identify any race-related variance with the limited number of semi-Mexican rootstocks included in our selection.

The use of rootstocks is a fundamental part of sustainable integrated pest management [47]. Moreover, as early as 1926, Webber stated that "no factor of the avocado industry is more important than rootstocks" [48]. Currently, several avocado rootstocks with tolerance to PRR are available commercially [16]. In olive, where VW greatly affects production, resistant rootstocks are available for VW management [35,36]. Grafting on resistant rootstocks to manage VW is also evaluated in several vegetable species [32,34]. Due to the variance found in this work for rootstocks susceptibility, it is reasonable to expect that avocado VW can be managed by using tolerant rootstocks. Moreover, the rootstocks in the current study were evaluated in field conditions under high disease pressure and for a substantial period (five years), allowing the assumption that the tolerance found should sustain over time in commercial orchards.

## 4. Conclusions

Performances of the rootstocks growing in a *V. dahliae-*infested orchard was highly variable, demonstrating that genetic variance may be used for the management of avocado VW. Planting unsuitable rootstocks (e.g., VC152) in contaminated soil increases the likelihood of tree loss during orchard establishment and low tree productivity in the long run. In contrast, the use of tolerant rootstocks (e.g., VC804) restricts VW symptoms and minimizes tree loss. However, further research is required for understanding the mechanisms of avocado tolerance to VW.

## Figures and Tables

**Table 1:** Evaluated rootstocks.

Rootstock	Race (Putative) ^a^	Propagation Method	Origin
VC26	W.I.	Clonal	A. Ben-Ya’acov collection; Israel
VC27	W.I.	Clonal	A. Ben-Ya’acov collection; Israel
VC28	W.I.	Clonal	A. Ben-Ya’acov collection; Israel
VC55	W.I.	Clonal	A. Ben-Ya’acov collection; Israel
VC66	W.I.	Clonal	A. Ben-Ya’acov collection; Israel
VC68	W.I.	Clonal	A. Ben-Ya’acov collection; Israel
VC96	W.I.	Clonal	A. Ben-Ya’acov collection; Israel
VC140	W.I.	Clonal	A. Ben-Ya’acov collection; Israel
VC152	W.I.	Clonal	A. Ben-Ya’acov collection; Israel
VC159	W.I.	Clonal	A. Ben-Ya’acov collection; Israel
VC162	W.I.	Clonal	A. Ben-Ya’acov collection; Israel
VC207	W.I.×Mex.	Clonal	Day; U.S.
VC320	W.I.	Clonal	Kaiima Bio Agritech; Israel
VC801	W.I.	Clonal	A. Ben-Ya’acov collection; Israel
VC802	W.I.	Clonal	A. Ben-Ya’acov collection; Israel
VC804	W.I.	Clonal	A. Ben-Ya’acov collection; Israel
VC840	Mex.	Clonal	A. Ben-Ya’acov collection; Israel
Latas	Mex.×Gu.	Clonal	Westfalia Fruit; South Africa
Dusa	Mex.×Gu.	Clonal	Westfalia Fruit; South Africa
Waldin	W.I.	Seed	U.S.
Degania 62	W.I.	Seed	A. Ben-Ya’acov collection; Israel
Degania 189	W.I.	Seed	A. Ben-Ya’acov collection; Israel
Nachlat 3	W.I.	Seed	A. Ben-Ya’acov collection; Israel

^a^ West Indian: W.I., Mexican: Mex., Guatemalan: Gu.

**Table 2:** Rootstocks performance.

Rootstock	Number of Evaluated Trees	Productivity (Fruit Tree^−1^) ^a^	Susceptibility (Index) ^b^	Performance Score ^c^
VC804	30	206 a	0	206
Dusa	30	213 a	20	193
Degania 189	30	176 abc	10	166
VC159	30	222 a	57	165
VC320	30	199 ab	40	159
Waldin	30	175 abc	17	158
Degania 62	30	160 abc	13	147
VC840	30	200 ab	57	143
VC207	30	169 abc	27	142
VC802	30	171 abc	40	131
VC68	30	166 abc	43	123
VC96	12	140 abc	25	115
VC801	30	132 abc	33	99
VC140	30	167 abc	70	97
VC55	30	129 abc	33	96
VC27	18	118 abc	50	68
VC162	6	136 abc	83	53
Nachlat 3	30	89 c	53	36
VC28	30	96 bc	63	33
VC26	26	98 abc	92	6
VC66	30	87 c	87	0
VC152	30	82 c	97	−15
Latas	12	0	158	−158

^a^ Accumulative number of fruits per tree harvested during seasons 2015–2018 (Figure 2d). Different letters indicate statistically significant difference according to Tukey–Kramer HSD test (*p* ≤ 0.05). ^b^ Percentage of tree loss (Figure 2a) + percentage of biotic stress symptoms (Figure 2b). ^c^ Productivity value minus the susceptibility index.
